# An Integrative Analysis of Preeclampsia Based on the Construction of an Extended Composite Network Featuring Protein-Protein Physical Interactions and Transcriptional Relationships

**DOI:** 10.1371/journal.pone.0165849

**Published:** 2016-11-01

**Authors:** Daniel Vaiman, Francisco Miralles

**Affiliations:** 1 INSERM, U1016, Institut Cochin, Paris, France; 2 CNRS, UMR8104, Paris, France; 3 Université Paris Descartes, Sorbonne Paris Cité, Paris, France; 4 DHU Risques et Grossesse, PRES Sorbonne Paris, Paris, France; Otto von Guericke Universitat Magdeburg, GERMANY

## Abstract

Preeclampsia (PE) is a pregnancy disorder defined by hypertension and proteinuria. This disease remains a major cause of maternal and fetal morbidity and mortality. Defective placentation is generally described as being at the root of the disease. The characterization of the transcriptome signature of the preeclamptic placenta has allowed to identify differentially expressed genes (DEGs). However, we still lack a detailed knowledge on how these DEGs impact the function of the placenta. The tools of network biology offer a methodology to explore complex diseases at a systems level. In this study we performed a cross-platform meta-analysis of seven publically available gene expression datasets comparing non-pathological and preeclamptic placentas. Using the rank product algorithm we identified a total of 369 DEGs consistently modified in PE. The DEGs were used as seeds to build both an extended physical protein-protein interactions network and a transcription factors regulatory network. Topological and clustering analysis was conducted to analyze the connectivity properties of the networks. Finally both networks were merged into a composite network which presents an integrated view of the regulatory pathways involved in preeclampsia and the crosstalk between them. This network is a useful tool to explore the relationship between the DEGs and enable hypothesis generation for functional experimentation.

## Introduction

Preeclampsia (PE) is a pregnancy condition characterized by hypertension and proteinuria. PE affects 5 to 7% of all pregnancies and remains a major cause of maternal morbidity and mortality. The disease constitutes also a major threat for the lives of child, being both a cause of prematurity and growth retardation; reviewed by [1, 2]. PE can develop at any time after 20 weeks of gestation, however early onset disease is more severe than later onset disease and associated with poorer outcomes for both mother and child. The pathogenesis of PE is thought to originate from the placenta since its delivery generally resolves the syndrome and remains the only cure. A consensus exists that in PE abnormal vascularization of the placenta leads to poor perfusion. Then, placental ischemia would cause intermittent hypoxia, oxidative stress, cell death, and the release to the maternal circulation of anti-angiogenic factors and debris that promote a systemic endothelial dysfunction [1–3]. To investigate the molecular mechanisms involved in this disease several studies have used genome-wide microarray expression analysis to identify differentially expressed Genes (DEGs) between the preeclamptic and the non-pathologic placenta; reviewed in [4]. Several DEGs have been found systematically modified in the preeclamptic placenta, including: LEP, FLT1, ENG, INHA [5–8]. Yet the characterization of the transcriptome signature of the preeclamptic placenta has not been followed by a detailed understanding on how the modifications in the expression of these genes impacts the function of the placenta. Biological processes in normal or pathological conditions results of complex interactions between genes, proteins, metabolites and other molecules. Systems biology approaches attempt to gain a deeper understanding of biological processes by integrating the ensemble of its components through the mathematical modelling of networks. In these networks, the components (genes/proteins/metabolites) of the system are represented as nodes and their interactions as edges [9]. Network analysis detects the relationships between its components and enables the physical or functional relationships between them to be interrogated. This methodology is useful to dissect the complexity of a biological process, providing information relevant to understand the process, including the pathways and the prioritization of genes involved in it [10]. Herein, we performed a cross-platform meta-analysis of several public gene expression datasets to identify DEGs that are consistently modified in PE. Then we used these DEGs to build a composite extended network integrating physical protein interactions (PPIs) as well as regulatory interactions with transcriptional factors (TFs). This composite extended network can be useful to investigate and gain insight into the molecular mechanisms involved in PE, to identify new biomarkers or potential drug targets.

## Materials and Methods

### Meta-analysis of microarray datasets

We performed a meta-analysis of several published gene expression data in preeclampsia. We searched the Gene Expression Omnibus (GEO) repository (http://www.ncbi.nlm.nih.gov/geo), to identify microarray datasets that compared gene expression in preeclamptic versus normal placentas. The keywords: preeclampsia, placenta, microarrays and gene-expression, were used for this search. This way, we identified a total of 12 microarray studies in the GEO repository. However, to be included in our study the microarray experiments had to carried out with RNAs obtained from placental biopsies collected at delivery and at relatively comparable gestational ages (30–39 weeks), include a minimal number of samples (≥ 5) and be comparable in terms of the microarray technology used. Thus finally, seven datasets were found meeting our criteria and considered eligible for our study, Table 1 [11–17].

**Table 1:** Datasets used for the meta-analysis.

Geo accesion	CO*	PE*	Unique gene IDs	Platform	Reference
GSE10588	26	17	16752	ABI Human Genome Survey Microarray V2	[11]
GSE14722	11	12	13292	Affymetrix Human Genome U133A	[12]
GSE24129	8	8	17100	Affymetrix Human Gene 1.0 ST	[13]
GSE25906	37	23	24870	Illumina human-6 V2.0	[14]
GSE44711	8	8	31329	Illumina human HT-12 V4.0	[15]
GSE43942	7	5	19767	NimbleGen Homo sapiens HG18 090828 HX12	[16]
GSE54618	12	12	31332	Illumina human HT-12 V4.0	[17]

* number of control (CO) or preeclampsia (PE) samples.

Since these studies used different microarray platforms, we performed our meta-analysis using the rank product algorithm implemented in the online INMEX software (http://inmex.ca), [18]. Before the datasets were analyzed, we used the processing modules (utilities) implemented in the INMEX software to annotate all probe identities from each dataset as Entrez gene IDs (for data consistency), transform the intensity values for gene expression to log_2_ and perform quantile normalization. A list of 369 differentially expressed genes (DEGs) were identified based on adjusted p-value (threshold was set at adjP < 0.05) and average fold change (FC ≥ 1.3 and FC≤ -1.3). FC were calculated as preeclampsia (PE) versus control (CO) samples. In addition, genes found to be significantly deregulated but showing discordant fold change in at least two of the seven datasets analyzed were discarded. Correction for multiple testing was done using Benjamin Hochberg False Discovery Rate (FDR).

### Enrichment analysis

The enrichment analysis of the DEGs was performed with the WebGestalt (http://bioinfo.vanderbilt.edu/webgestalt) bioinformatics resource [19]. WebGestalt (WEB-based GEne SeT AnaLysis Toolkit) incorporates updated information from different public resources and provides an easy way to make sense out of gene lists. Databases interrogated include: Gene Ontology (GO), Kyoto Encyclopedia of Genes and Genomes (KEGG), and WikiPathways [20–22]. The significance of the detected enrichments was calculated using the Benjamini & Hochberg multiple test adjustment. In addition, WebGestalt was also used to perform enrichment analysis of the network modules identified by community clustering.

### Extended Protein-Protein Interaction networks: Construction and Analysis

The DEGs identified in our meta-analysis were used as seeds to build a network of protein-protein interactions (PPIs). We constructed a network that not only consists of the seed proteins but also of their direct PPI neighbors and the interactions between these proteins (first order network). We obtained high confidence PPIs from the Biological General Repository for Interaction Datasets (BioGRID), (http://thebiogrid.org), [23]. The PPI network was constructed, visualized and analyzed using the Cytoscape 3.2.1 software and its complementary applications [24]. The network was analyzed for both centrality and modularity parameters. The centrality parameters of the networks were analyzed using the Cytoscape application NetworkAnalyzer [25]. Two topological parameters Betweenness Centrality (BC) and node degree were used to identify hub genes [26]. In PPI networks genes or proteins are represented by nodes and the interactions between the nodes are represented as edges. BC is defined as the number of shortest paths from all vertices to all others that pass through that node. BC reflects the amount of control that the node exerts over the interaction of other nodes in the network. Degree indicates the number of edges linked to a given node. Nodes with higher degree and high BC are hub genes which correspond to the most functionally relevant elements in the network. Modular analysis was performed using the fast-greedy HE (G) algorithm of the Community Clusters Glay implemented in the Cytoscape Apps [27]. The idea behind identifying functional modules is that proteins interacting with each other have higher probability of sharing the same function than none interacting proteins. Thus, finding functional modules in a biological network is similar to identify clusters of densely interacting nodes.

### Regulatory Network Construction

We built a regulatory network by extracting gene regulatory relationships from RegNetwork (http://www.regnetworkweb.org) an integrated database of transcriptional and post-transcriptional regulatory networks in human and mouse [28]. This database integrates the documented regulatory interactions among transcription factors (TFs), microRNAs (miRNAs) and target genes from 25 selected databases including: JASPAR, TRANSFAC, TRED and USCS [29–31] [32]. It also incorporates potential regulatory relationships based on TFs binding sites. The DEGs were used as seeds to extract high confidence transcriptional regulatory relationships from the human RegNetwork.

## Results

### Identification of DEGs by meta-analysis

Seven microarray datasets were extracted from the GEO database, which meet our criteria for meta-analysis (Table 1). Cross-platform meta-analysis of the selected studies using the rank product algorithm resulted in the identification of a total 1940 DEGs at adjP <0.05. Significant genes having the same direction of fold change in at least 5 out of 7 datasets and with an average log_2_FC ≥ 0.3 were considered for further analysis (The average log_2_FC being the average log_2_FC for every single gene for the 7 datasets). This resulted in a list of 369 DEGs, of which 212 were up-regulated in PE and 157 down-regulated genes in PE (S1 Table). Among the consistently up-regulated genes in PE with the higher mean log_2_FC and lowest p-value we detected in descending order LEP, FSTL3, PAPPA2, INHA, FLT1, SPAG4 and BHLHE40. The down-regulated genes with the largest mean log_2_FC and lowest p-value included CLDN1, SPP1, ACTG2, NR2F1, GCLM and MMP1. By analyzing the literature we found that several of the top DEGs identified in our meta-analysis have been validated experimentally as differentially expressed in preeclamptic placentas (S2 Table).

### Functional and pathway enrichment analysis

Enrichment analysis was performed to gain insight on the biological role of the DEGs. The most over-represented Gene Ontology (GO) terms in the biological process category included: “response to steroid hormone stimulus”, “response to wounding”, “cell migration”, “and response to lipid” and “cell proliferation”. In the category of GO cellular component most enriched terms were “extracellular region”, “extracellular matrix” and “plasma membrane”. A complete list of GO terms is provided in S3 Table. KEGG and WIKI pathways analysis revealed that the DEGs are associated with a number of significant pathways (Table 2).

**Table 2:** Significant KEGG and Wiki pathways associated with the DEGs.

**Pathways (KEGG)**	**Genes in DataSet**	**Genes in Pathway**	**adj P-Value**
Focal adhesion	19	200	1.62E-12
Pathways in cancer	20	326	5.81E-10
Metabolic pathways	33	1130	4.67E-08
Protein digestion and absorption	9	81	9.63E-07
ECM-receptor interaction	9	85	1.18E-06
Cytokine-cytokine receptor interaction	14	265	1.65E-06
Complement and coagulation cascades	7	69	3.41E-05
Arginine and proline metabolism	6	54	7.84E-05
Steroid hormone biosynthesis	6	56	8.10E-05
Cysteine and methionine metabolism	5	36	0.0001
Gap junction	6	90	0.0006
Purine metabolism	7	162	0.0023
MAPK signaling pathway	10	268	0.0006
P53 signaling pathway	5	68	0.0016
Tight junction	6	132	0.0042
Insulin signaling pathway	6	138	0.0048
Chemokine signaling pathway	7	189	0.0048
Endocytosis	7	201	0.0060
Regulation of actin cytoskeleton	7	213	0.0079
**Pathways (Wiki)**	**Genes in DataSet**	**Genes in Pathway**	**adj P-Value**
Adipogenesis	14	130	6.93E-10
Corticotropin-releasing hormone	10	123	3.49E-06
Selenium Pathway	8	108	6.53E-05
Oxidative Stress	5	30	6.70E-05
TGF-Beta Signaling Pathway	6	61	0.0001
Prostaglandin Synthesis and Regulation	4	31	0.0006
Senescence and Autophagy	7	120	0.0006
Metapathway biotransformation	8	177	0.0010
Cytochrome P450	5	65	0.0010

These included: “focal adhesion”, “pathways in cancer”, “metabolic pathways”, and “cytokine-cytokine receptor interaction”. “Focal adhesion” and “regulation of actin cytoskeleton” were detected in both the KEGG and Wiki pathway databases. The most significantly deregulated signaling pathways included: MAPK, P53, TGF-Beta, and Chemokine signaling. To complement the functional analysis we calculated the histogram of gene frequency in the different pathways identified by the enrichment analysis (S1 Fig). Thus, TGFB1 and PDGFRA are the genes with the higher frequencies. They are present in 8 and 7 pathways respectively. CYP11A1 and SERPINE1 belong to 6 pathways, FOS, IGF1 and PIK3CB are present in 5 pathways. COL1A1 and CYP11A1 are present in 4 pathways. The other genes present lower frequencies. 26 genes were present in 3 pathways, 32 genes in 2 pathways and 64 genes belong only to 1 pathway.

### Construction of a physical PPI network

To interpret the functional meaning of the identified DEGs at the protein level, we constructed a physical PPI network for the proteins encoded by these genes. Physical PPI interactions were retrieved from the BioGRID database [23]. We constructed a network that not only consists of the seed proteins but also their direct PPI neighbors (first order interaction network) and the interactions between these proteins. The proteins (nodes) and resulting interactions (edges) were imported into Cytoscape for visualization and further analysis [24]. This resulted in a network composed 1231 proteins and 5486 interactions. The network contained 214 out of the 369 DEGs identified in the meta-analysis. For the remaining DEGs no high confidence PPIs were reported in the BioGRID database. Biological networks have topological characteristics distinguishing them from random networks [26]. Among these the node degree distribution usually follows a power law. As shown (S2A Fig), this is the case for our network: R^2^ = 0.913. This also means that a few nodes are highly connected (hubs) while a majority of nodes interact only with one or a few neighbors. The hub nodes play a major role in the architecture of the network and usually correspond to proteins of special biological relevance in the system under study. Here we used two topological parameters to identify hub proteins: betweenness centrality (BC) and degree (both defined in the methods section). These parameters were calculated using the Cytoscape NetworkAnalyzer app. The topological scores (for the top 50 genes) are supplied in S4 Table. When considering the BC parameter, the principal hub genes include EGFR, ZBTB16, GRB2, EP300, TP53 and GOLGA2. Several of the DEGs are present among the top 50 hub genes including ZBTB16, CASK, FOS, BHLHE40, TGFB1 and NOS3.

### Module detection using connectivity patterns

The PPI network was further subdivided into connectivity modules using the fast-greedy HE (G) algorithm of the Community Clusters Glay [27]. This resulted in the identification of nine connectivity modules closely connected. The Modules were analyzed for functionality enrichment using the gene ontology (GO) database (Table 3).

**Table 3:** Enriched GO terms in the modules detected by the GLAY community clustering algorithm.

Module	GO ID	GO Term	Observed Genes	N° genes	Adj P-value
**Module 1**	GO:0006357	Transcription From Rna Polymerase II Promoter	133	292	9.52E-58
	GO:0009893	Positive Regulation Of Metabolic Process	145		5.58E-51
	GO:0000989	Transcription Factor Binding Transcription Factor Activity	60		5.06E-31
	GO:0044428	Nuclear Part	171		1.41E-56
**Module 2**	GO:0051171	Regulation Of Nitrogen Compound Metabolic Process	70	255	0.0603
	GO:0019219	Regulation Of Nucleobase-Containing Compound Metabolic Process	68		0.0603
	GO:0045111	Intermediate Filament Cytoskeleton	23		4.46E-12
**Module 3**	GO:0007167	Enzyme Linked Receptor Protein Signaling Pathway	77	219	6.32E-38
	GO:0007169	Transmembrane Receptor Protein Tyrosine Kinase Signaling Pathway	67		6.32E-38
	GO:0005829	Cytosol	74		2.07E-12
	GO:0005615	Extracellular Space	42		2.07E-12
**Module 4**	GO:0051270	Regulation Of Cellular Component Movement	18	180	0.0006
	GO:0005615	Extracellular Space	25		7.31E-05
**Module 5**	GO:0006915	Apoptotic Process	16	67	0.0051
	GO:0065008	Regulation Of Biological Quality	22		0.0051
	GO:0044449	Contractile Fiber Part	10		5.09E-09
	GO:0005737	Cytoplasm	50		7.32E-06
**Module 6**	GO:0050817	Coagulation	11	64	0.0004
	GO:0009611	Response To Wounding	15		0.0009
	GO:0001071	Nucleic Acid Binding Transcription Factor Activity	11		0.0162
**Module 7**	GO:0001819	Positive Regulation Of Cytokine Production	7	55	0.0007
	GO:0031349	Positive Regulation Of Defense Response	7		0.0012
	GO:0044459	Plasma Membrane Part	14		0.0194
	GO:0005615	Extracellular Space	8		0.0350
**Module 8**	GO:0007264	Small Gtpase Mediated Signal Transduction	11	35	2.50E-05
	GO:0045184	Establishment Of Protein Localization	14		5.72E-05
	GO:001503	Protein Transport	13		0.0002
	GO:0007154	Cell Communication	23		0.0010
**Module 9**	GO:0042060	Wound Healing	6	22	0.0059
	GO:0007596	Blood Coagulation	5		0.0059
	GO:0040011	Locomotion	8		0.0059

Thus, the bigger module, module1 was composed of 292 nodes and significantly enriched in the biological processes terms: positive regulation of transcription from RNA polymerase II promoter (adjP = 9.52e-58), positive and regulation metabolic process activity (adjP = 5.58e-51). The smaller module, module 9, contained 22 nodes and was associated with healing (adjP = 0.0059) and coagulation processes (adjP = 0.0059).

### Construction of a transcription factor regulatory network

We used RegNetwork [28] a database of transcriptional and post-transcriptional regulatory networks, to extract regulatory interactions among the 369 DEGs of the preeclampsia meta-signature and transcription factors (TF). Regulatory relationships experimentally validated and predicted were considered. This generated a network composed of 571 nodes and 2214 regulatory interactions. Among the nodes, 365 corresponded to DEGs identified in the meta-analysis and the remaining 206 were inferred TFs. Among the DEGs we identified 30 genes encoding TFs and regulating other DEGs. Topological analysis of the network using Cytoscape NetworkAnalyzer shows that the node degree distribution follows a power law (R^2^ = 0.841); (S2B Fig). The TFs with the higher out degree (number of regulatory interactions), included the SP1, POU2F1, JUN, CREB1, MYC, CEBPA, TFAP2A, ARNT, NFKB1, GATA1, STAT1, TP53, ATF2 and HIF1A. Among the DEGs encoding TFs those with the highest out degree were: CEBPA, TFAP2A, FOS, SREBF1, JUNB, FOSB and NR2F1. Several of the TFs with a higher out degree can bind to the top DEGs identified in our meta-analysis. For example SP1 binds to transcription factor binding sites (TFBS) in the promoter of SPAG4, SPP1 or BCL6; CEBPA binds to the promoter of LEP, PAPPA2, FLT1 and BHLHE40; POU2F1 has TFBS in the promoters of down-regulated genes such as CLDN1, SPP1 or ACTG2. Interestingly and in accordance with the role of hypoxia in preeclampsia, HIF1A can regulate the expression of several top DEGs identified in the meta-analysis including: LEP, FLT1, INHA, BHLHE40, SPAG4, HK2, HILPDA and ERO1L.

### Composite interactions PE network

**Fig 1:**
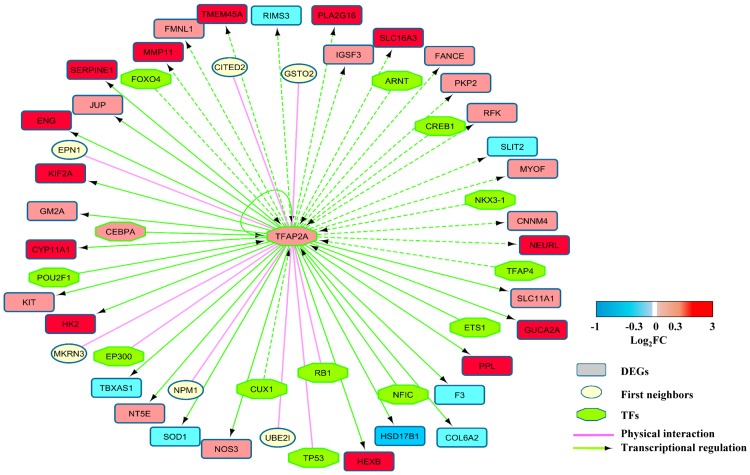
The interactions of AP2A in the composite extended network. The proteins encoded by the DEGs identified by the meta-analysis are shown as ellipses colored in red when up-regulated or blue if down-regulated. First neighbors of the proteins encoded by the DEGs are show as circles colored in yellow. Transcription factors are shown as octagons colored in green (or red/blue if they belong to the DEGs). Physical interactions are depicted in mauve and regulatory interactions in green. The regulatory interactions are shown as a continuous line when validated experimentally or as a discontinuous line when predicted on the basis of the presence of a transcription binding site (TFBS) in the target gene.

The physical interactions PPI Network and the TFs-DEGs regulatory network were merged using Cytoscape to generate a composite network which integrates both kinds of interactions. Thus we obtained a network composed of 1542 nodes and 7718 edges. To illustrate it, Fig 1 shows a portion of the composite network displaying the transcription factors AP2A, its target among the DEGs, its physical protein interactions and its transcriptional regulators. For visualization purposes TFs were differentiated from their targets or other proteins in the network using a different symbol. Regulatory interactions were colored in green and physical interactions in mauve. The mean log_2_FC of the DEGs was incorporated in the network to visualize up or down-regulation. The complete composite network is provided as supplementary data in form of an XML file which can be visualized and explored using Cytoscape.

## Discussion

Herein we have performed a cross-platform meta-analysis of seven gene-expression datasets comparing non-pathological and preeclamptic placentas. Using a rank product algorithm we identified 369 DEGs consistently modified across the considered datasets. In a previous study we used a vote counting strategy which led to the identification of only 98 DEGs in preeclamptic placentas [8]. Sixty one DEGs identified in our previous study were also identified in the present study. Vote counting is a simple method for summarizing evidence from multiple datasets; however it does not take into account the quality of the studies, the size of the samples, or the size of the effect. In the present study we used a rank product algorithm, a simple non-parametric statistical method based on ranks of fold changes [33]. Methods combining P-values or effect sizes have the disadvantage that the results can be often dominated by outliers, a significant problem when thousands of genes are compared in the noisy environment of microarray experiments. The methods combining rank statistics can be used to alleviate this problem. Comparative studies suggest that the rank product method outperforms the other methods in terms of sensitivity and specificity, especially in a setting of small sample size and large between-study variation [34]. In addition to our studies, two other meta-analysis on transcriptomic datasets of preeclamptic placentas have been conducted recently [5, 6]. The study by Moslehi and coworkers was conducted on 4 datasets using a combining P-values method and identified a total of 419 DEGs. The overlap with the present study shows 139 common DEGs. The second one published by van Uitert and coworkers, was conducted on 11 placenta gene expression datasets which were preprocessed to conduct quality analysis. A combining size effect algorithm using inverse variance random effects was applied, resulting in the identification of 388 DEGs. In this case the overlap with the current study is of 148 common DEGs. Discrepancies between the studies can be attributed to the different number of datasets and different statistics meta-analysis methodologies.

Functional enrichment analysis of the DEGs identified in the present study, revealed that they are mainly related to biological functions such as Focal adhesion, metabolic pathways, ECM-interactions, cytokine-cytokine interactions, complement and coagulation cascades, etc. All these processes have been previously associated with preeclampsia [3]. To further characterize the relationship among the DEGs we attempted to identify physical interactions among the proteins encoded by the DEGs. However, the scarcity of direct interactions between the DEGs imposed the need to create a first order network. Thus we build an extended network which includes not only the interactions between the DEGs but also the interactions between the DEGs and their first neighbors. This resulted in a network composed of 1231 proteins and 5486 physical interactions. To evaluate the biological significance of proteins in a complex disease such as PE, network biology considers the topological position of a protein as crucial as can be any eventual modification in its expression level. Within a network not all nodes are equally important. Many studies have shown that “hub” nodes are more likely related to diseases [35, 36]. Proteins occupying a “hub” position are potentially key molecules in signaling, as they are interacting with many other proteins, being thus able to receive and transmit multiple signals [37]. Herein proteins with high node degree and betweenness centrality were considered “hubs”. Among the top 50 hubs we found EGFR, ZBTB16, GRB2, EP300, TP53 and GOLGA2. Several of the DEGs are present among the top 50 hubs including ZBTB16, CASK, FOS, BHLHE40, TGFB1 and NOS3. Below we discuss the role of some of these hubs in PE.

EGFR encoding the epidermal growth factor receptor is the principal hub of our network. This protein is a receptor for members of the epidermal growth factor (EGF) family. In addition to EGF this family includes the transforming growth factor–alpha (TGFA), heparin-binding EGF-like growth factor (HBEGF), betacellulin (BTC), amphiregulin (AREG) and epiregulin (EREG). Accumulating evidence suggests that human trophoblasts survival and invasive capacities are linked to these growth factors. EGF protects cultured human cytotrophoblats against induced apoptosis [38, 39]. Also *in vitro* studies have shown that cytotrophoblast motility and invasiveness are stimulated by TGFA and heparin-binding EGF-like growth factor HBEGF [40, 41]. HBEGF is involved in extravillous trophoblast differentiation and it also protects cytotrophoblast cells from apoptosis when exposed to hypoxia or oxidative stress consecutive to hypoxia/reoxygenation [42]. A recent study has shown that in vitro, EGF, TGFA, BTC and EREG can also prevent apoptosis in cytotrophoblast exposed to hypoxia/reoxygenation stress [43]. Decreased expression of EGF, TGFA and HBEGF has been reported in the preeclamptic placenta, while expression of BTC, EREG and AREG show no significant differences [43, 44]. BTC and EREG could at least in part compensate the lack for the other EGF family members since they are effective in preventing cytotrophoblast apoptosis. However, the analysis of EGFR mRNA expression in the placenta has revealed two different full-length transcripts and one truncated transcript, p110/EGFR. In the preeclamptic placenta two independent studies have found significantly higher mRNA expression of the truncated transcript, while there were no significant differences in the mRNA expression of the two full-length transcripts [43, 45]. Since the p110/EGFR isoform contains an extracellular domain and lacks the intracellular domain it should not be able to signal upon EGF-binding and thus could act as a dominant negative. The high expression of the p110/EGFR in the preeclamptic placenta would contribute to impair the EGF signaling system [43]. Thus, concordantly with our bioinformatics analysis experimental evidence support an important role for EGFR in the development of PE. Our study is also in good consonance with two previous studies. A first study by Tejera and coworkers identified EGFR and GRB2 genes as central components of the PE network [46]. Also, an integrative analysis by Moleshi and coworkers identified EGFR signaling deficiency combined to hypoxia/oxidative stress as central mechanisms of PE [5].

Among the DEGs the transcriptional repressor ZBTB16 (PLZF) appears as one of the principal hubs in the network. ZBTB16 is a member of the kruppel-like zinc finger protein family involved in a multitude of biological processes including spermatogenesis, hematopoiesis, myeloid differentiation lymphoid development, cytokine production, programming and maturation of NKT and INKT cells, cellular proliferation and apoptosis [47, 48]. Due to its role in limiting cell-cycle progression and cellular proliferation PLZF is known as a tumor suppressor [49]. Recruitment of co-repressors and subsequent chromatin remodeling has been shown to underlie the repressor function of ZBTB16 [48, 50]. Although considered as a repressor an increasing number of studies describe ZBTB16 as an activator of transcription [48]. A role of ZBTB16 in the control of inflammation is emerging. ZBTB16 would form a co-repressor complex with HDAC3 and NF-κB in order to moderate the inflammatory program [51, 52]. A possible function of ZBTB16 in placenta physiopathology remains to be explored, however a recent study linked it to hydatidiform molar pregnancies through its physical interaction with NLRP7 [53]. According to this study NLRP7 (a protein involved in innate immunity and apoptosis) could trap ZBTB16 in the cytoplasm and disrupt its nuclear inhibitory function, and this would lead to excessive trophoblastic proliferation. Interestingly ZBTB16 is known to interact physically with the EGFR ligand HBEGF [54]. The EGFR ligands, are synthesized as type I transmembrane protein precursors and are expressed on the plasma membrane [55]. Membrane-anchored HBEGF, is cleaved from the plasma membrane in a process termed ectodomain shedding, which yields soluble HBEGF (sHBEGF) and a carboxy-terminal fragment of HBEGF (HBEGF-CTF). The sHBEGF binds to the EGFR and induces EGFR phosphorylation and subsequent activation of downstream signaling events. In parallel, the HBEGF-CTF translocates to the nucleus, where it induces nuclear export of the transcriptional repressors ZBTB16 and BCL6, thereby facilitating cell cycle progression [54, 56, 57]. The ectodomain shedding is done by disintegrin and metalloproteinase (ADAM) members. A recent study has shown that TGFβ induces HBEGF shedding and EGFR transactivation through ADAM17 activation in gastric cancer cells [58]. It would be interesting to investigate if a similar mechanism is also at work in trophoblasts since aberrant TGFβ signaling is involved in PE [3].

We identified BHLHE40 (also known as STRA13, DEC1) as another of the DEGs occupying a hub position. This gene has been found systematically up-regulated in the preeclamptic placenta [5, 6, 8]. BHLHE40 is a transcriptional repressor binding as an homodimer to class B E-box sites with high affinity; reviewed by [59, 60]. Its transcriptional repression activity includes recruitment of co-repressors (such HDAC1) that mediated repressive chromatin marks or methylate non-histone proteins; dimerization with bHLH factors to form non-functional complexes; competition for binding to E-box sites; interaction with non-bHLH transcription factors to inhibit their activity; and promotion of proteasome-mediated degradation. BHLHE40 is involved in different biological processes and its expression is up-regulated by numerous stimuli including retinoic acid, serum deprivation, TGFβ, cAMP, light, cytokines, insulin and hypoxia. BHLHE40 regulates the differentiation of several cell types, thus gain and loss of function experiments suggest that it can repress mesoderm differentiation and enhance muscle regeneration after injury [61, 62]. BHLHE40 is deregulated in a wide variety of cancers and impairs the DNA mismatch repair mechanism by repressing the expression of MLH-1 [63]. Many studies have shown that BHLHE40 mediates cell cycle arrest and promotes cellular senescence [60]. It has been proposed that its effects on cell cycle could result of down-regulation of c-Myc, and cyclin D1 as well as its interaction with p53 [64, 65]. Its capacity to induce senescence seems to be partially p53 dependent [66]. Also, depending on the cell type BHLHE40 has been shown to either induce or block apoptosis [59]. Hypoxia has been identified as a hallmark of the preeclamptic placenta. Interestingly, BHLHE40 is a target of the hypoxia inducible factor (HIF1) and is induced by hypoxia [67–69]. Under normoxic conditions, BHLHE40 expression is down-regulated by von Hippel-Lindau (VHL) a component of the VHL/E3 ligase complex which ubiquitinates and targets HIF1 for degradation [70]. Recent studies indicate that BHLHE40 could act also as a regulator of HIF1 and hypoxia responses. Thus, induction of HIF-1α and VEGF through an EGFR/PI3K/BHLHE40 pathway has been reported [71]. As an effector of HIF1 it has been shown that BHLHE40 regulates adipogenesis through a mechanism involving recruitment of HDAC1 to repress CEBPB transcriptional activity [72]. Also, under hypoxic conditions BHLHE40 represses sterol regulatory element binding protein 1c (SREBP1c), involved in the induction of several enzymes of the lipogenesis pathway [73]. The control exerted by BHLHE40 on wide range of biological functions, its role in mediating hypoxia responses, and is systematic up-regulation in the preeclamptic placenta suggests that it could play an important role in this disease. It is likely that exploring the function of BHLHE40 in cytotrophoblasts under normoxia and hypoxia could reveal new cues on the molecular mechanisms at work in the preeclamptic placenta.

## Conclusions

In this study we have used publically available microarray datasets comparing gene expression in preeclamptic versus non-pathologic placentas. Other studies analyzing global gene expression in the preeclamptic placenta have been published but the datasets are not publically accessible. Thus, the number of datasets constitutes a first limitation of our study which could have reached stronger value if more datasets were included. Another limitation of the present study comes from the coverage and quality of the human interactome data. Despite the best curation efforts, the interactome remains incomplete, current coverage is estimated at 20% and biased toward much-studied proteins [74–76]. In principle the incompleteness of the human PPI network poses limitation to any study of network properties in the context of normal physiology or disease. However, a recent study showed that notwithstanding its incompleteness, the available interactome has sufficient coverage to pursue a systematic network-based approach to human diseases [77].

Our composite network summarizes current knowledge on the relationships between gene/proteins involved in PE. It constitutes a model which can be easily upgraded with new PPI or regulatory interactions.

## Supporting Information

S1 Fig The histogram of gene frequency in the pathways associated with the DEGs. The histogram shows how often a single DEG identified in the pathway enrichment analysis appears in the different pathways.
(PPTX)

S2 Fig The node degree distribution of the extended physical PPI (A) and TFs regulatory networks (B). In both cases the node degree distribution satisfy a power law distribution in the form y = a_*_x^-b^.
(PPTX)

S1 Network The composite extended network build using the 369 DEGs identified in the meta-analysis as seeds. Cytoscape XML file.
(ZIP)

S1 Table The 369 preeclampsia differentially expressed genes (DEGs) identified by the meta-analysis. (XLSX)

S2 Table List of genes identified as differentially expressed in preeclampsia in our meta-analysis and whose differential expression has been experimentally validated by other studies. (DOCX)

S3 Table GO terms “Biological process” and “Cellular Component” associated with the DEGs identified by the meta-analysis. (DOCX)

S4 Table The top 50 hubs in the PPI network based on their betweenness centrality (BC) values. (DOCX)
