# Supplementary material for: An Integrative Analysis of Preeclampsia Based on the Construction of an Extended Composite Network Featuring Protein-Protein Physical Interactions and Transcriptional Relationships
Source: PLoS One. 2016 Nov 1;11(11):e0165849. doi: 10.1371/journal.pone.0165849 (PMC5089765; doi:10.1371/journal.pone.0165849)
Supplement: S1 Fig — The histogram shows how often a single DEG identified in the pathway enrichment analysis appears in the different pathways. (PPTX) [file pone.0165849.s001.pptx]

## Slide 1
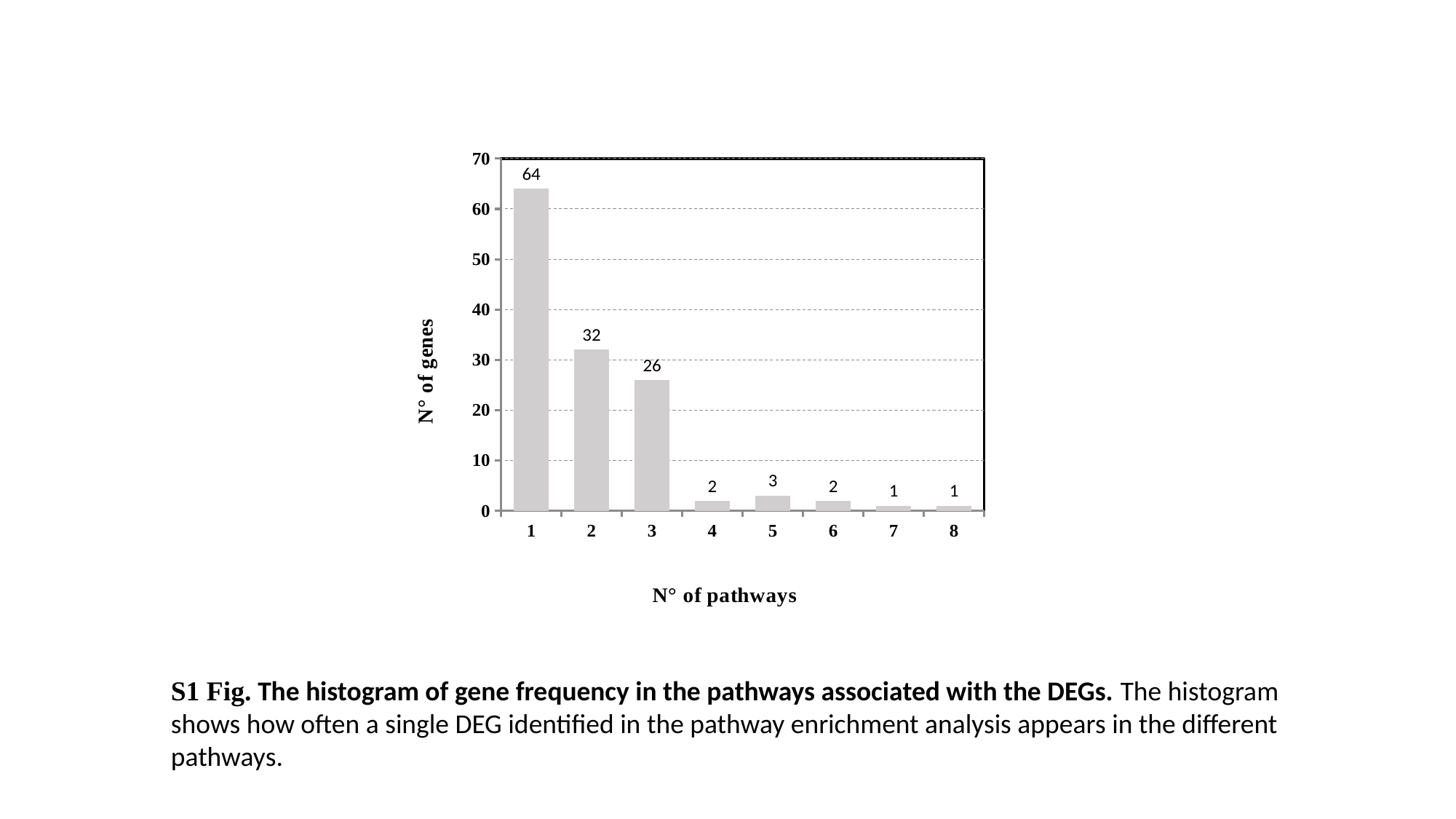

### Chart
| Category | Fréquence |
|---|---|S1 Fig. The histogram of gene frequency in the pathways associated with the DEGs. The histogram shows how often a single DEG identified in the pathway enrichment analysis appears in the different pathways.
