# Supplementary material for: An Integrative Analysis of Preeclampsia Based on the Construction of an Extended Composite Network Featuring Protein-Protein Physical Interactions and Transcriptional Relationships
Source: PLoS One. 2016 Nov 1;11(11):e0165849. doi: 10.1371/journal.pone.0165849 (PMC5089765; doi:10.1371/journal.pone.0165849)
Supplement: S2 Fig — In both cases the node degree distribution satisfy a power law distribution in the form y = a*x-b. (PPTX) [file pone.0165849.s002.pptx]

## Slide 1
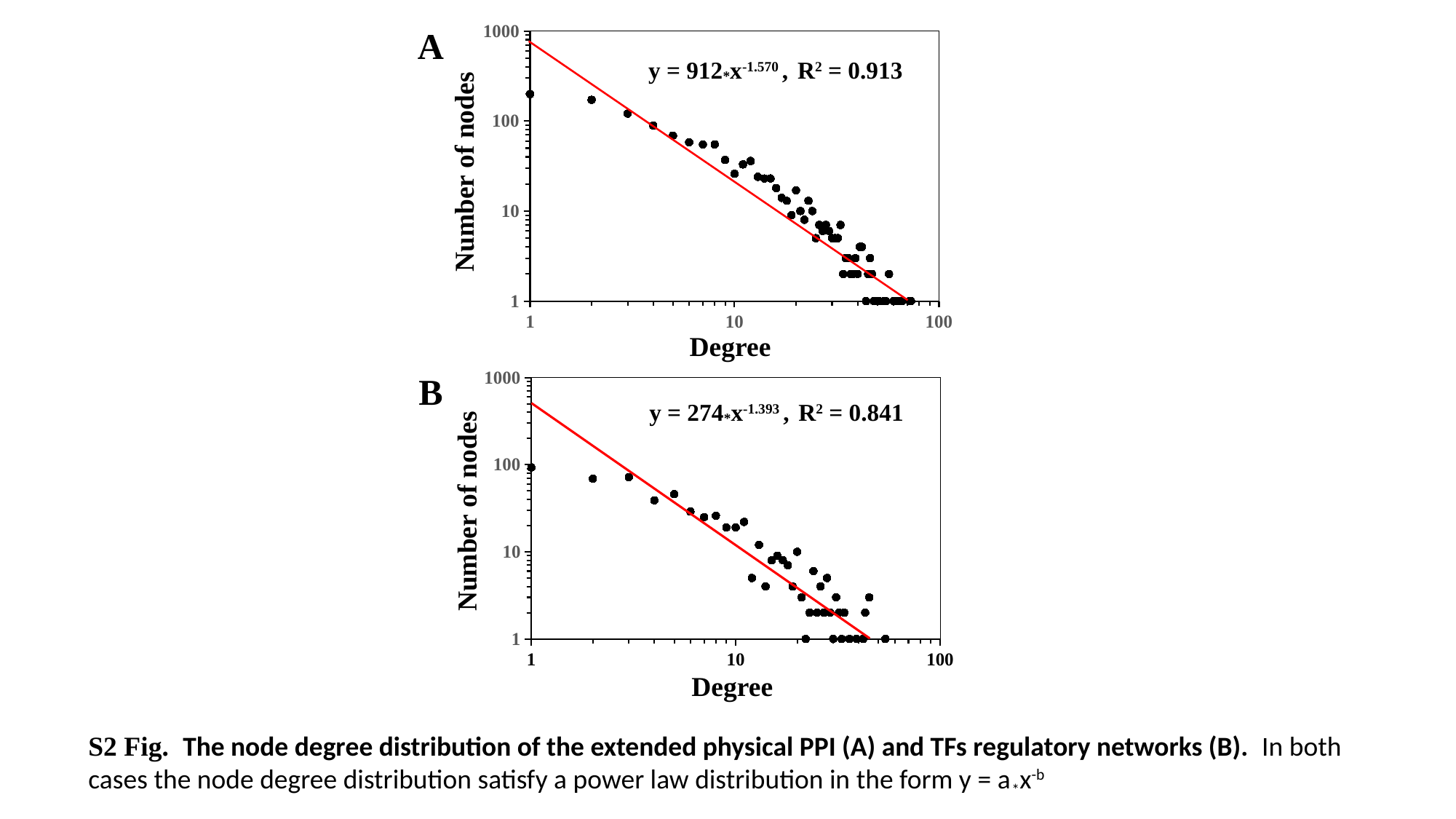

### Chart
| Category | |
|---|---|A
y = 912*x-1.570 , R2 = 0.913
Number of nodes
Degree
### Chart
| Category | Degree |
|---|---|B
y = 274*x-1.393 , R2 = 0.841
Number of nodes
Degree
S2 Fig. The node degree distribution of the extended physical PPI (A) and TFs regulatory networks (B). In both cases the node degree distribution satisfy a power law distribution in the form y = a*x-b
