# Supplementary material for: An Integrative Analysis of Preeclampsia Based on the Construction of an Extended Composite Network Featuring Protein-Protein Physical Interactions and Transcriptional Relationships
Source: PLoS One. 2016 Nov 1;11(11):e0165849. doi: 10.1371/journal.pone.0165849 (PMC5089765; doi:10.1371/journal.pone.0165849)
Supplement: S2 Table — (DOCX) [file pone.0165849.s005.docx]

**Table S2. List of genes differentially expressed in preeclampsia according to the present meta-analysis, whose differential expression has been experimentally validated by other studies.**

**References**

1. Enquobahrie DA, Meller M, Rice K, Psaty BM, Siscovick DS, Williams MA. Differential placental gene expression in preeclampsia. Am J Obstet Gynecol. 2008;199(5):566 e1-11.

2. Meng T, Chen H, Sun M, Wang H, Zhao G, Wang X. Identification of differential gene expression profiles in placentas from preeclamptic pregnancies versus normal pregnancies by DNA microarrays. OMICS. 2012;16(6):301-11.

3. Winn VD, Gormley M, Paquet AC, Kjaer-Sorensen K, Kramer A, Rumer KK, et al. Severe preeclampsia-related changes in gene expression at the maternal-fetal interface include sialic acid-binding immunoglobulin-like lectin-6 and pappalysin-2. Endocrinology. 2009;150(1):452-62.

4. Xiang Y, Cheng Y, Li X, Li Q, Xu J, Zhang J, et al. Up-regulated expression and aberrant DNA methylation of LEP and SH3PXD2A in pre-eclampsia. PLoS One. 2013;8(3):e59753.

5. Varkonyi T, Nagy B, Fule T, Tarca AL, Karaszi K, Schonleber J, et al. Microarray profiling reveals that placental transcriptomes of early-onset HELLP syndrome and preeclampsia are similar. Placenta. 2011;32 Suppl:S21-9.

6. Sitras V, Paulssen RH, Gronaas H, Leirvik J, Hanssen TA, Vartun A, et al. Differential placental gene expression in severe preeclampsia. Placenta. 2009;30(5):424-33.

7. Pryor-Koishi K, Nishizawa H, Kato T, Kogo H, Murakami T, Tsuchida K, et al. Overproduction of the follistatin-related gene protein in the placenta and maternal serum of women with pre-eclampsia. BJOG. 2007;114(9):1128-37.

8. Centlow M, Wingren C, Borrebaeck C, Brownstein MJ, Hansson SR. Differential gene expression analysis of placentas with increased vascular resistance and pre-eclampsia using whole-genome microarrays. J Pregnancy. 2011;2011:472354.

9. Hoegh AM, Borup R, Nielsen FC, Sorensen S, Hviid TV. Gene expression profiling of placentas affected by pre-eclampsia. J Biomed Biotechnol. 2010;2010:787545.

10. Lee GS, Joe YS, Kim SJ, Shin JC. Cytokine-related genes and oxidation-related genes detected in preeclamptic placentas. Arch Gynecol Obstet. 2010;282(4):363-9.

11. Jebbink J, Keijser R, Veenboer G, van der Post J, Ris-Stalpers C, Afink G. Expression of placental FLT1 transcript variants relates to both gestational hypertensive disease and fetal growth. Hypertension. 2011;58(1):70-6.

12. Kaartokallio T, Cervera A, Kyllonen A, Laivuori K, Group FCI. Gene expression profiling of pre-eclamptic placentae by RNA sequencing. Sci Rep. 2015;5:14107.

13. Lievano S, Alarcon L, Chavez-Munguia B, Gonzalez-Mariscal L. Endothelia of term human placentae display diminished expression of tight junction proteins during preeclampsia. Cell Tissue Res. 2006;324(3):433-48.

14. Kawasaki K, Kondoh E, Chigusa Y, Ujita M, Murakami R, Mogami H, et al. Reliable pre-eclampsia pathways based on multiple independent microarray data sets. Mol Hum Reprod. 2015;21(2):217-24.

15. Lian IA, Toft JH, Olsen GD, Langaas M, Bjorge L, Eide IP, et al. Matrix metalloproteinase 1 in pre-eclampsia and fetal growth restriction: reduced gene expression in decidual tissue and protein expression in extravillous trophoblasts. Placenta. 2010;31(7):615-20.

16. Nishizawa H, Ota S, Suzuki M, Kato T, Sekiya T, Kurahashi H, et al. Comparative gene expression profiling of placentas from patients with severe pre-eclampsia and unexplained fetal growth restriction. Reprod Biol Endocrinol. 2011;9:107.

17. Trifonova EA, Gabidulina TV, Ershov NI, Serebrova VN, Vorozhishcheva AY, Stepanov VA. Analysis of the placental tissue transcriptome of normal and preeclampsia complicated pregnancies. Acta Naturae. 2014;6(2):71-83.

18. Louwen F, Muschol-Steinmetz C, Friemel A, Kampf AK, Tottel E, Reinhard J, et al. Targeted gene analysis: increased B-cell lymphoma 6 in preeclamptic placentas. Hum Pathol. 2014;45(6):1234-42.

19. Rumer KK, Uyenishi J, Hoffman MC, Fisher BM, Winn VD. Siglec-6 expression is increased in placentas from pregnancies complicated by preterm preeclampsia. Reprod Sci. 2013;20(6):646-53.

20. Nishizawa H, Pryor-Koishi K, Kato T, Kowa H, Kurahashi H, Udagawa Y. Microarray analysis of differentially expressed fetal genes in placental tissue derived from early and late onset severe pre-eclampsia. Placenta. 2007;28(5-6):487-97.

21. Toft JH, Lian IA, Tarca AL, Erez O, Espinoza J, Eide IP, et al. Whole-genome microarray and targeted analysis of angiogenesis-regulating gene expression (ENG, FLT1, VEGF, PlGF) in placentas from pre-eclamptic and small-for-gestational-age pregnancies. J Matern Fetal Neonatal Med. 2008;21(4):267-73.

22. Tsai S, Hardison NE, James AH, Motsinger-Reif AA, Bischoff SR, Thames BH, et al. Transcriptional profiling of human placentas from pregnancies complicated by preeclampsia reveals disregulation of sialic acid acetylesterase and immune signalling pathways. Placenta. 2011;32(2):175-82.

23. Venkatesha S, Toporsian M, Lam C, Hanai J, Mammoto T, Kim YM, et al. Soluble endoglin contributes to the pathogenesis of preeclampsia. Nat Med. 2006;12(6):642-9.

24. Mayor-Lynn K, Toloubeydokhti T, Cruz AC, Chegini N. Expression profile of microRNAs and mRNAs in human placentas from pregnancies complicated by preeclampsia and preterm labor. Reprod Sci. 2011;18(1):46-56.

25. Petsas G, Jeschke U, Richter DU, Minas V, Hammer A, Kalantaridou S, et al. Aberrant expression of corticotropin-releasing hormone in pre-eclampsia induces expression of FasL in maternal macrophages and extravillous trophoblast apoptosis. Mol Hum Reprod. 2012;18(11):535-45.

26. Lapaire O, Grill S, Lalevee S, Kolla V, Hosli I, Hahn S. Microarray screening for novel preeclampsia biomarker candidates. Fetal Diagn Ther. 2012;31(3):147-53.
