# Supplementary material for: An Integrative Analysis of Preeclampsia Based on the Construction of an Extended Composite Network Featuring Protein-Protein Physical Interactions and Transcriptional Relationships
Source: PLoS One. 2016 Nov 1;11(11):e0165849. doi: 10.1371/journal.pone.0165849 (PMC5089765; doi:10.1371/journal.pone.0165849)
Supplement: S3 Table — (DOCX) [file pone.0165849.s006.docx]

**S3 Table. GO terms Biological Process and Cellular Component associated with the DEGs identified by the meta-analysis**
