# Supplementary material for: An Integrative Analysis of Preeclampsia Based on the Construction of an Extended Composite Network Featuring Protein-Protein Physical Interactions and Transcriptional Relationships
Source: PLoS One. 2016 Nov 1;11(11):e0165849. doi: 10.1371/journal.pone.0165849 (PMC5089765; doi:10.1371/journal.pone.0165849)
Supplement: S4 Table — (DOCX) [file pone.0165849.s007.docx]

**S4 Table. The top 50 hubs in the PPI network based on their betweenness centrality (BC) values**
